# CARERS, AGE, AND A NONINTERSECTIONAL FEMINIST DISCOURSE: THE (IN)VISIBLITY OF CARE IN US LITERARY FICTION

**DOI:** 10.1093/geroni/igae098.0877

**Published:** 2024-12-31

**Authors:** Eva-Maria Trinkaus

**Affiliations:** University of Klagenfurt, Austria, Klagenfurt, Karnten, Austria

## Abstract

How do we want to be cared for and who do we want our carers to be? In this narrative analysis of US literary fiction, I am looking at intersectional inequality in the context of domestic work as care work (Folbre 2006). Focusing on overlapping aspects of aging studies and postcolonial studies, I trace care work in literary fiction back to the late 19th and early 20th century to look into the feminist shift of ‘new women’ leaving the domestic spheres to participate in a broader public discourse and thereby opening up a gap for ‘other’ workers to fill within the setting of care. In order to understand what constitutes a ‘good care’ or a ‘good care worker’ respectively, this contribution investigates the fictional representation of caregivers in literary texts in order to look at how intersections of multilayered marginalizing aspects – gender, race, class, age, education, among others (cf. Crenshaw 1991, Calasanti & Slevin 2002) – contribute to the undervaluing and under-recognition of care work, but do not necessarily affect its quality (cf. D’Ignazio & Klein 2023). By investigating dimensions of care work through literary fiction, this contribution aims at challenging the current esteem of care work, and contributes to a broader discussion of adding visibility and value to the professional field.

